# Prospektive, multizentrische Untersuchung des Outcomes nach 12 Monaten beim komplexen regionalen Schmerzsyndrom

**DOI:** 10.1007/s00482-024-00837-7

**Published:** 2024-09-28

**Authors:** H. Hofbauer, A. Brinkmann, E. Maurer, B. Weber, G. Hänle, P. Steffen

**Affiliations:** 1 Sektion Schmerztherapie, Klinik für Anästhesiologie und Intensivmedizin, Universitätsklinikum Ulm, Albert-Einstein-Allee 23, 89081 Ulm, Deutschland; 2 Klinik für Anästhesie, operative Intensivmedizin und spezielle Schmerztherapie, Klinikum Heidenheim, Schloßhaustraße 100, 89522 Heidenheim, Deutschland; 3 Buchenstraße 39, 72517 Sigmaringendorf, Deutschland; 4 Abteilung für Gynäkologie und Geburtshilfe, Robert-Bosch-Krankenhaus, Auerbachstraße 110, 70376 Stuttgart, Deutschland

**Keywords:** Komplexes regionales Schmerzsyndrom, Therapieergebnis, Chronischer Schmerz, Leitlinien, Physiotherapie, Complex regional pain syndrome, Treatment outcome, Chronic pain, Guidelines, Physical therapy

## Abstract

**Hintergrund und Fragestellung:**

Das komplexe regionale Schmerzsyndrom („complex regional pain syndrome“ [CRPS]) kann langfristig zu starken Schmerzen und eingeschränkter Funktionalität führen. Leitlinien sollen helfen, die Behandlungsabläufe zu optimieren. Es sollte untersucht werden, welches Outcome unter leitliniengestützter Therapie nach 1 Jahr erreicht wird.

**Material und Methoden:**

In einer prospektiven Multicenterstudie wurde bei 40 Patient*innen mit neu diagnostiziertem CRPS untersucht, wie sich die Schmerzen und Funktionseinschränkungen innerhalb von 1 Jahr veränderten. Zudem wurde untersucht, inwieweit der Zeitpunkt der Diagnosestellung sowie invasive Maßnahmen Einfluss auf diese Outcomeparameter haben.

**Ergebnisse:**

Alle Patient*innen erhielten Physio- und/oder Ergotherapie, eine Therapie mit Glukokortikoiden und/oder Bisphosphonat 29 (72,5 %), diverse invasive Maßnahmen erfolgten bei 13 (32,5 %). Nach 1 Jahr waren sowohl die Schmerzen als auch die Funktion signifikant verbessert, zwei Drittel berichteten über eine erträgliche mittlere Schmerzstärke. Eine schwere Funktionseinschränkung nach Von-Korff-Disability-Punkten fand sich nach 1 Jahr bei 9 (22,5 %), eine mittel- bzw. schwergradige Einschränkung nach ärztlicher Einschätzung bei 6 (15 %) bzw. 3 (7,5 %) Betroffenen. Eine frühere Diagnosestellung und ein entsprechend früherer Therapiebeginn korrelierten mit einem besseren Outcome bzgl. Schmerzen und Funktionalität nach von Korff, jedoch nicht nach ärztlicher Einschätzung. Der Einfluss invasiver Verfahren auf die Outcomeparameter war gering.

**Diskussion:**

Eine an den Leitlinien orientierte Therapie führte mehrheitlich zu einem guten Outcome bzgl. Schmerzen und Funktionalität. Eine frühzeitige Diagnosestellung korrelierte mit besserem Outcome, somit sollten Verdachtsfälle zügig einer Einrichtung mit entsprechender Expertise zugeführt werden.

**Zusatzmaterial online:**

Die Online-Version dieses Beitrags (10.1007/s00482-024-00837-7) enthält den CRPS-Erfassungs- und -Behandlungsbogen.

## Abstract (en)

**Background and objectives:**

Complex regional pain syndrome (CRPS) can lead to severe pain and limited functionality in the long term. Guidelines should help to optimize treatment procedures. It should be investigated which outcome is achieved after 1 year with guideline-based therapy.

**Materials and methods:**

In a prospective multicenter study, 40 patients with newly diagnosed CRPS were examined to determine how their pain and functional limitations changed within 1 year. In addition, it was investigated whether the time of diagnosis and invasive measures influence these outcome parameters.

**Results:**

All patients received physiotherapy and/or ergotherapy, treatment with glucocorticoids and/or bisphosphonates 29 (72.5%); various invasive measures were carried out in 13 (32.5%). After 1 year, both pain and functionality were significantly improved; two-thirds reported a tolerable average pain intensity. Severe functional impairment according to von Korff disability points was found after 1 year in 9 (22.5%), and a moderate or severe impairment according to medical evaluation in 6 (15%) and 3 (7.5%) patients, respectively. Earlier diagnosis and corresponding earlier start of treatment correlated with better outcome in terms of pain and functionality according to von Korff, but not according to medical evaluation. The influence of invasive procedures on the outcome parameters tended to be low.

**Conclusion:**

Guideline-based treatment led to a good outcome in terms of pain and functionality in the majority of patients. Early diagnosis correlated with better outcome, so suspected cases should be referred quickly to a medical facility with appropriate expertise.

## Hintergrund

Das komplexe regionale Schmerzsyndrom („complex regional pain syndrome“ [CRPS]) geht mit teils erheblicher Funktionsbeeinträchtigung und starken Schmerzen einher. Man unterscheidet hierbei das CRPS Typ I ohne vom CRPS Typ II mit nachweisbarer Nervenverletzung. Eine kausale Therapie existiert nicht, in den Leitlinien [3, 5] finden sich aber zahlreiche Empfehlungen; vor allem Physiotherapie mit „graded exposure“ und ergotherapeutische Behandlungen inkl. Spiegeltherapie [26] gelten als essenziell. Medikamentös werden neben klassischen (Ko‑)Analgetika als spezifischere Substanzen Glukokortikoide, Bisphosphonate und Dimethylsulfoxid (DMSO) empfohlen. Invasive Maßnahmen sollen im Behandlungsalgorithmus zurückhaltend eingesetzt und nur durch spezialisierte Zentren durchgeführt werden.

Ziel dieser multizentrischen Untersuchung war es, Verlauf und Outcome hinsichtlich Schmerzen und Funktionseinschränkung nach 1 Jahr leitliniengestützter Behandlung bei Patient*innen mit neu diagnostiziertem CRPS zu erheben. Berücksichtigt wurde dabei auch der Zeitraum zwischen Auftreten erster Symptome und Diagnosestellung bzw. Zugang zu adäquater Schmerztherapie, um zu überprüfen, ob ein früher Therapiebeginn das Outcome verbessert. Außerdem sollte der Einfluss etwaiger invasiver Maßnahmen untersucht werden.

## Material und Methoden

### Studiendesign

Die prospektive, multizentrische klinische Beobachtungsstudie zu Therapiestrategien und -erfolg bei CRPS erfolgte in den Schmerzambulanzen von vier Kliniken (Uniklinik Ulm [UL; Anzahl Patient*innen mit neu diagnostiziertem CRPS pro Jahr: 45–50 Fälle], SRH-Kliniken Sigmaringen [SIG; 20–25 Fälle/Jahr], Klinikum Heidenheim [HDH; 5–10 Fälle/Jahr], Klinik am Eichert, Göppingen [GP, 40–50 Fälle/Jahr]). Eingeschlossen wurden Patient*innen, bei denen von den teilnehmenden Studienärzt*innen der Zentren neu die Diagnose eines CRPS I oder II entsprechend den „Budapest-Kriterien“ [11, 12] gestellt wurde. Aufgrund der Rahmenbedingungen konnte nicht immer ein konsekutiver Studieneinschluss der neuen Fälle gewährleistet werden (v. a. bei notfallmäßiger und somit zeitlich sehr beschränkter Konsilvorstellung z. B. durch chirurgische Abteilungen ohne vorherige Terminvergabe; nicht alle Ärzt*innen der beteiligten Zentren nahmen aktiv an der Studie teil, sodass deren Patient*innen nicht eingeschlossen wurden). Spezifische Therapien des CRPS (z. B. Kortisonstoßtherapie, Bisphosphonate) waren bis zur Aufnahme in die Untersuchung nicht erfolgt, wenngleich nichtspezifische Maßnahmen wie z. B. Gabe von nichtsteroideale Antirheumatika (NSAR) und Physiotherapie bereits durchgeführt worden waren.

Bei Erstdiagnose (Zeitpunkt 0) sowie nach 1, 2 und 12 Monaten wurden Befund- und Behandlungsbögen ausgefüllt (vgl. Online-Zusatzmaterial). Die Behandlung orientierte sich an den zum Erfassungszeitraum gültigen Leitlinien der Deutschen Gesellschaft für Neurologie (DGN) von 2008 [3]. Ausgewertet wurden neben der Schmerzstärke CRPS-typische Veränderungen, durchgeführte übende Maßnahmen, eingesetzte Medikamente und ggf. die Effektivität invasiver Therapien bezogen auf Schmerzstärke und Funktionseinschränkung. Beim Ersttermin wurde der Deutsche Schmerzfragebogen, Version 2007, an den Folgeterminen der Schmerzverlaufsfragebogen der Deutschen Schmerzgesellschaft ausgefüllt. Aus diesen wurden die Von-Korff-Disability-Punkte ausgewertet [16]. Zusätzlich wurde von ärztlicher Seite jeweils die Funktionseinschränkung erhoben: 0 = keine Einschränkung; 1 = leichte Einschränkung, voll berufs-/alltagstauglich; 2 = deutliche Einschränkung, deutliche Einschränkung im Berufsalltag; 3 = schwere Einschränkung, auf Hilfe im Alltag angewiesen.

Der Einschluss in die Studie erfolgte nach Aufklärung und schriftlicher Einwilligung. Ein positives Votum der für alle Kliniken zuständigen Ethikkommission der Universität Ulm lag vor. Die Studie wurde nach den Grundsätzen der Deklaration von Helsinki (Revision 2008) durchgeführt.

### Statistische Auswertung

Zur Feststellung eines Mittelwertunterschieds wurden der t‑Test und Post-hoc-Test (Bonferroni-Korrektur oder Greenhouse-Geisser-Korrektur) für abhängige bzw. unabhängige Stichproben sowie der Mann-Whitney-U-Test für unabhängige Stichproben verwendet. Um den Einfluss einer oder mehrerer unabhängiger Variablen auf abhängige Variablen zu prüfen, wurden Varianzanalysen vorgenommen. Zusammenhänge der Variablen wurden mittels Spearman-Korrelationskoeffizient ermittelt (Spearmans Rho).

Die statistischen Verfahren wurden mit IBM® SPSS Statistics 17.0.0 angewandt. Es wurde eine α‑Fehlerwahrscheinlichkeit von 5 % festgelegt.

## Ergebnisse

41 Patient*innen mit nach Budapest-Kriterien neu diagnostiziertem CRPS wurden zwischen 07/2010 und 02/2014 eingeschlossen. Ein Patient wünschte die Therapie vorzeitig abzubrechen und wurde aus der Studie ausgeschlossen. Die ausgewerteten 40 Datensätze verteilten sich auf die teilnehmenden Kliniken wie folgt: SIG 23, UL 9, GP 5, HDH 3. In dieser Erhebung waren 62,5 % der Teilnehmenden weiblich. In 95 % lag ein CRPS I vor, wobei meist die obere Extremität betroffen war (Tab. 1). In 5 Fällen lag der Symptombeginn bis zum Zeitpunkt der Erstdiagnose länger als 6 Monate zurück. Vor Beginn des CRPS waren 14 Betroffene arbeitslos oder bezogen Rente. 46,2 % der zuvor Berufstätigen waren auch nach 1 Jahr noch nicht wieder arbeitsfähig.

**Tab. 1:** Daten zu Demografie und CRPS-Erkrankung

Alter	M = 55,8 Jahre (31–82 Jahre)
Geschlecht	♀ 25 (62,5 %), ♂ 15 (37,5 %)
Diagnosestellung nach Symptombeginn	M = 83,2 Tage, Median 73 Tage (3–300 Tage)
Auslöser	Elektive Operation: *n* = 8 (20 %) Trauma ohne anschließende Operation: *n* = 9 (22,5 %) Trauma mit Operation: *n* = 21 (52,5 %) Kein Trauma/Auslöser erinnerlich: *n* = 2 (5 %)
CRPS-Typ	CRPS I: *n* = 38 (95 %), CRPS II: *n* = 2 (5 %)
Lokalisation	Obere Extremität: *n* = 29 (72,5 %), untere Extremität: *n* = 11 (27,5 %)

### Medikamentöse und invasive Therapiemaßnahmen

Die während des Beobachtungszeitraums eingesetzte Medikation ist in Tab. 2 dargestellt.

**Tab. 2:** Im Untersuchungszeitraum eingesetzte Medikation

Medikamente	Nichtopioide	Opioide WHO II	Opioide WHO III	Koanalgetika	Glukokortikoide oral	Bisphosphonate	DMSO
Anzahl Pat. *n* (%)	34 (85 %)	15 (38 %)	2 (5 %)	20 (50 %)	16 (40 %)	25 (62,5 %)	15 (38 %)

(Nichtopioide: Metamizol, NSAR, Coxibe; Opioide WHO-Stufe II: Tilidin/Naloxon; Opioide WHO-Stufe III: Hydromorphon, Tapentadol; Koanalgetika: Pregabalin, Gabapentin, Trizyklika)

16 Patient*innen (40,0 %) erhielten orale Glukokortikoide, 25 (62,5 %) Bisphosphonate (intravenös oder oral), 12 (30 %) beides sowie eine Patientin Kortikoidsalbe lokal neben einer Bisphosphonatgabe. Keine Verordnung einer der beiden Substanzgruppen erfolgte in 11 Fällen (27,5 %). Die Therapieregime unterschieden sich bei der oralen Glukokortikoidgabe mit im Durchschnitt 31 Anwendungstagen (17–62 Tage) sowie insbesondere bei den Bisphosphonaten hinsichtlich verwendeter Substanz und Anwendungsdauer (Pamidronsäure 60 mg bzw. Zoledronsäure 4 mg als intravenöse Einmalgabe sowie Alendronsäure oral im Mittel über 72 Tage [13–272 Tage]). Alle erhielten Physio- und/oder Ergotherapie. Bei 39 Patient*innen (97,5 %) erfolgte eine krankengymnastische Behandlung, 33 (82,5 %) erhielten Lymphdrainage (jeweils im Mittel 19 Wochen). Ergotherapie erfolgte bei 24 (60 %), Spiegeltherapie bei 25 (62,5 %).

Invasive Verfahren wurden bei 13 Patient*innen (32,5 %) eingesetzt. In 9 Fällen (22,5 %) erfolgte eine Regionalanalgesie mit niedrig konzentriertem Lokalanästhetikum über Katheter (6 × Plexus brachialis über supraklavikulären oder axillären Zugang, 2 × Periduralkatheter, 1 × N. ischiadicus) im Durchschnitt für 10,2 Tage (0–18 Tage). Ein Patient zog sich akzidentell den supraklavikulären Katheter am ersten Tag und erhielt noch eine Ganglion-stellatum-Blockade. In 4 weiteren Fällen (10 %) erfolgten Serien von Ganglion-stellatum-Blockaden (*n* = 8–15 Blockaden).

### Schmerzen und Funktionseinschränkung

**Abb. 1:**
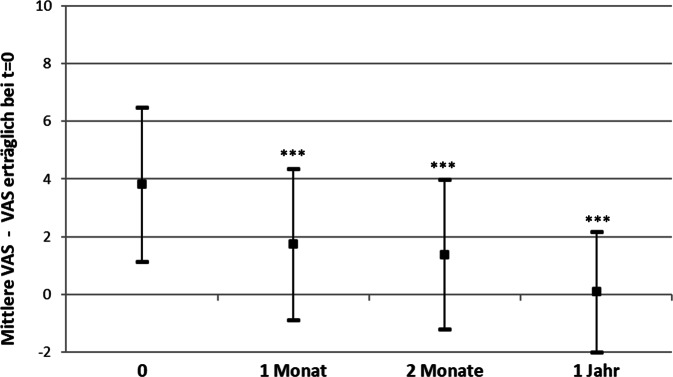
Differenz zwischen der mittleren zum Zeitpunkt t = 0 als erträglich eingestuften Schmerzstärke (mittlere VAS – VAS erträglich bei t = 0; M ± SD); ***p < 0,001

Zum Zeitpunkt t = 0 wurde die als erträglich eingestufte Schmerzstärke nach VAS (visuelle Analogskala) als Ziel einer suffizienten Schmerzeinstellung erhoben. Die Differenz der mittleren Schmerzstärke zu den einzelnen Untersuchungszeitpunkten zu diesem als erträglich angegebenen Zielwert wurde erfasst (Abb. 1). Der berichtete Rückgang für die Differenz war signifikant (F (3, 114) = 35,614, *p* ≤ 0,001; ${\eta }_{p}^{2}$ = 0,477; einfaktorielle Varianzanalyse für abhängige Stichproben). Dieser signifikante Rückgang war für alle Messzeitpunkte hochsignifikant (nach 1 Monat: Post-hoc-Test, Bonferroni, ***p ≤ 0,001, 95 %-Intervallgrenzen: 0,948; 3,202; nach 2 Monaten: Post-hoc-Test, Bonferroni, ***p ≤ 0,001, 95 %-Intervallgrenzen: 1,284; 3,566; nach 1 Jahr: Post-hoc-Test, Bonferroni, ***p ≤ 0,001, 95 %-Intervallgrenzen: 2,588; 4,862). Der zu Beginn als erträgliches Schmerzniveau angegebene Wert wurde nach 1 Jahr bei 67,5 % der Fälle (*n* = 27) erreicht bzw. unterschritten, bezogen auf die maximale VAS bei 52,5 % (*n* = 21). Somit konnte aber nach 1 Jahr bei einem Drittel (bezogen auf die mittlere VAS) und knapp der Hälfte (bezogen auf die maximale VAS) der Patient*innen nicht ein erträgliches Schmerzniveau erreicht werden.

**Abb. 2:**
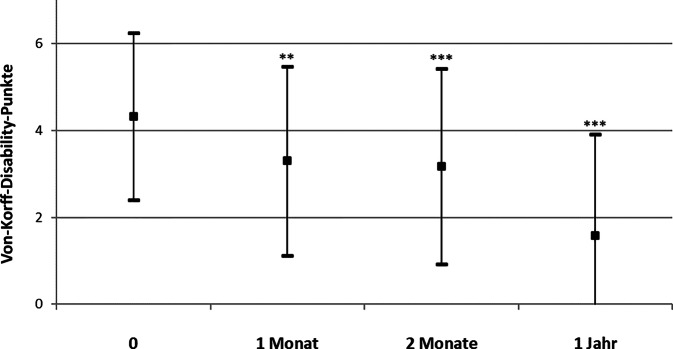
Von-Korff-Disability-Punkte (5–6 = starke Beeinträchtigung, 3–4 = mäßige Beeinträchtigung, < 3 = leichte Beeinträchtigung; M ± SD); **p < 0,01, ***p < 0,001

Die Funktionseinschränkung erhoben mittels der Von-Korff-Disability-Punkte besserte sich ebenfalls signifikant (*X*^*2*^(3) = 38,279; *p* ≤ 0,001; Friedman-Test; für den Unterschied nach einem Monat **p ≤ 0,01; nach 2 Monaten ***p ≤ 0,001; nach einem Jahr ***p ≤ 0,001; Post-hoc-Test, Vorzeichentest, Abb. 2), wobei die Anzahl der Patient*innen mit schwerer Beeinträchtigung (Disability-Punkte 5 bzw. 6) von 23 (57,5 %) nach 1 Jahr auf 9 (22,5 %) fiel.

**Abb. 3:**
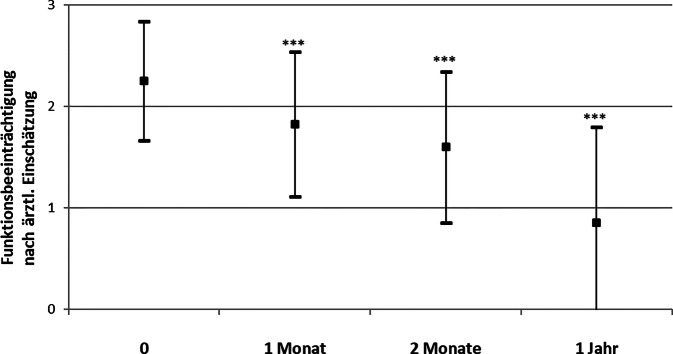
Funktionsbeeinträchtigung nach ärztlicher Einschätzung (Einschränkung: 3 = schwer, 2 = mittel, 1 = leicht [voll berufs-/alltagstauglich], 0 = keine; M ± SD); ***p < 0,001

Vergleichbar wurde die Funktionsbeeinträchtigung nach ärztlicher Einschätzung beurteilt, auch hier kam es zu einer Reduktion der beurteilten Funktionseinschränkung (*X*^*2*^(3) = 61,106; ***p ≤ 0,001; Friedman-Test, für alle Messzeitpunkte ***p ≤ 0,001; Post-hoc-Test, Vorzeichentest, Abb. 3). Zu Beginn wurden dabei alle Patient*innen als funktionseingeschränkt eingeschätzt (3 leicht, 24 mittel, 13 schwer), nach 1 Jahr noch 21 (12 leicht, 6 mittel, 3 schwer).

### Einfluss des Zeitpunkts der Diagnosestellung

**Abb. 4:**
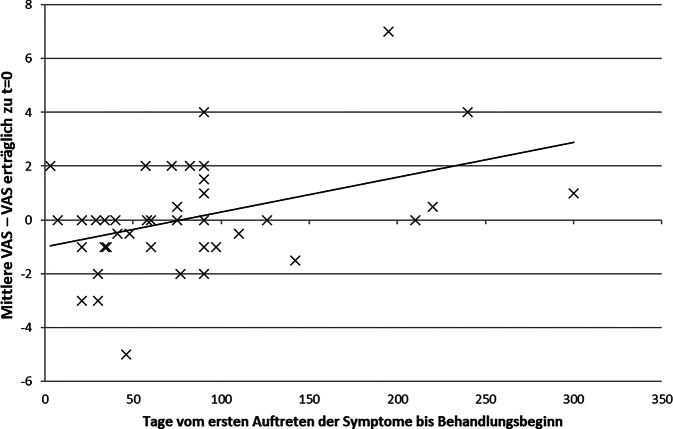
Einfluss des Behandlungsbeginns auf Therapieerfolg durch Korrelation des Schmerzes (mittlere VAS – VAS erträglich zu t = 0) nach 1 Jahr mit der Dauer vom ersten Auftreten der Symptome bis zum Behandlungsbeginn in Tagen

**Abb. 5:**
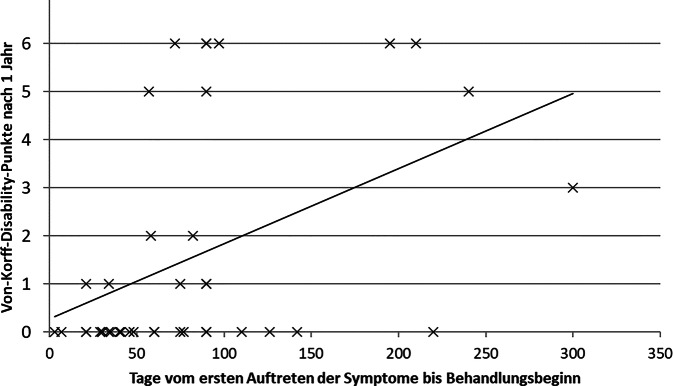
Einfluss des Behandlungsbeginns auf Therapieerfolg durch Korrelation der Von-Korff-Disability-Punkte nach 1 Jahr mit der Dauer vom ersten Auftreten der Symptome bis zum Behandlungsbeginn in Tagen

**Abb. 6:**
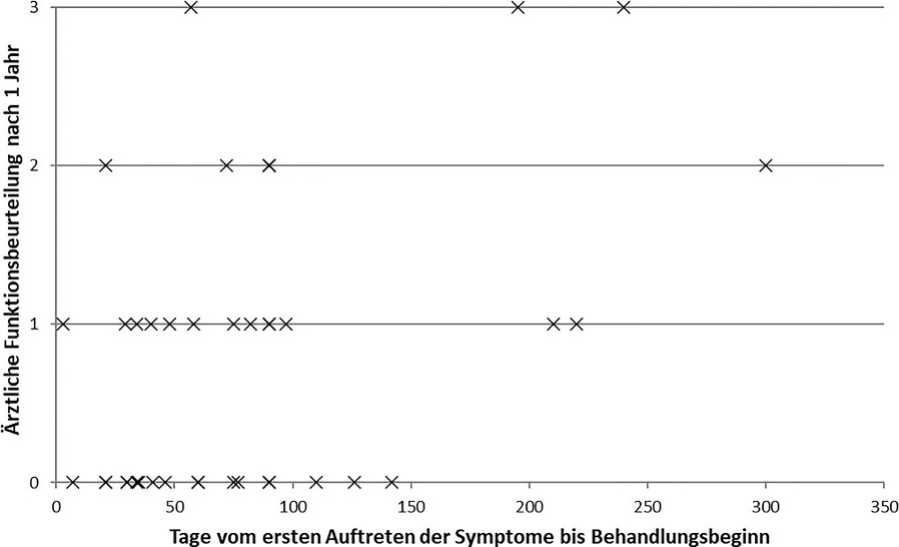
Einfluss des Behandlungsbeginns auf den Therapieerfolg durch Korrelation der Funktionsbeeinträchtigung nach ärztlicher Einschätzung mit der Dauer vom ersten Auftreten der Symptome bis zum Behandlungsbeginn in Tagen (3 = schwere Einschränkung, 2 = deutliche Einschränkung, 1 = leichte Einschränkung, 0 = keine Einschränkung)

Es zeigte sich bezogen auf Schmerzen eine signifikante Korrelation für ein besseres Outcome nach einem Jahr (Abb. 4), wenn eine Behandlung früh startete (Produkt-Moment-Korrelation, r = 0,414; *p* ≤ 0,01), ebenso bzgl. der Disability-Punkte nach von Korff (Abb. 5) für eine geringere Beeinträchtigung (Spearmans Roh, r = 0,449; *p* ≤ 0,01). Eine Korrelation hinsichtlich des Zeitpunkts der Diagnosestellung fand sich bei der ärztlichen Einschätzung der Funktionsbeeinträchtigung jedoch nicht (Abb. 6).

Invasive Maßnahmen beeinflussten das Outcome bezogen auf Schmerzstärke oder Funktionsbeeinträchtigung nach von Korff zu keinem Zeitpunkt, jedoch wurde nach ärztlicher Einschätzung zum Zeitpunkt 0 eine signifikant schlechtere generelle Funktion bei den später invasiv behandelten Patient*innen dokumentiert (Mann-Whitney-U-Test; *U* = 92,5; *N*_*i*_ = 13; *N*_*ni*_ = 27; **p ≤ 0,01; $\overline{x}_{{med_{i}}}$ = 26,88; $\overline{x}_{{med_{ni}}}$ = 17,43).

## Diskussion

Patient*innen, die an einem CRPS erkranken, sind oftmals langfristig aufgrund von Schmerzen und Funktionseinschränkungen beeinträchtigt. Im Rahmen dieser prospektiven Untersuchung sollte geprüft werden, wie sich das Krankheitsbild im Laufe eines Jahres bei leitlinienorientierter Therapie entwickelt. Die Diagnose erfolgte anhand der Budapest-Kriterien [11, 12], die Therapie sollte sich an den zum Studienzeitpunkt gültigen Leitlinien der DGN orientieren [3]. Weitergehende Vorgaben wurden nicht gemacht, da die aus Sicht des Klinikers notwendige Therapie zum Einsatz kommen sollte.

Das Durchschnittsalter beim Auftreten des CRPS lag mit 55,8 Jahren knapp 10 Jahre über dem Ergebnis von Sandroni et al. [24]. Vor Auftreten der Erkrankung waren bereits 18 % altersbedingt in Rente, insgesamt waren 35 % berentet oder arbeitslos.

Vergleichbar zu anderen Erhebungen [17] waren Frauen sowie die obere Extremität häufiger betroffen und es lag mehrheitlich ein CRPS Typ I vor. Die häufigste Ursache war, wie in anderen Untersuchungen [17, 24], die distale Radiusfraktur, gefolgt von anderen Frakturen und kleinen operativen Eingriffen.

Eine orale Glukokortikoid- und/oder Bisphosphonatgabe erfolgte in 28 Fällen mit unterschiedlichen Therapieregimen. Ein kürzlich publiziertes Review [2] bestätigt die Wirksamkeit von Glukokortikoiden bei CRPS, wobei keine Aussagen über notwendige Dosis, Anwendungsdauer und Applikation getroffen werden konnten. In 11 Fällen kamen weder Glukokortikoide noch Bisphosphonat zum Einsatz, z. B. wegen Kontraindikationen oder fehlenden Zeichen einer Inflammation. Zudem empfahl die Leitlinie von 2008 entgegen der aktualisierten Form von 2018 [5] die Bisphosphonatgabe nur nach Frakturen. Die sonstige eingesetzte Pharmakotherapie zur Behandlung der Schmerzen entsprach den Empfehlungen der Leitlinie und wurde an die jeweilige klinische Situation angepasst.

Physio- und/oder Ergotherapie als elementare Basismaßnahmen kamen immer zum Einsatz. Allerdings sind Qualitätsunterschiede in einem ambulanten Setting mit einer Vielzahl an Behandlern anzunehmen und könnten das Outcome beeinflusst haben. Ob spezifisch für CRPS empfohlene Maßnahmen wie „graded exposure“ und „graded motor imaging“ [4, 25, 26] angewendet wurden, wurde nicht erhoben. Der Einsatz von Spiegeltherapie wurde in 62,5 % dokumentiert. Leider ist im klinischen Alltag ein Setting mit vielen beteiligten Therapeuten in unterschiedlichen Einrichtungen der Regelfall, sodass die Behandlungsqualität abweichen kann. Ein interdisziplinäres Vorgehen mit einem festen, erfahrenen Behandlerteam und entsprechend abgestimmter Therapie ist im ambulanten Setting dagegen der selten umsetzbare Idealfall [14]. Auch in dieser Untersuchung war dies in keiner der beteiligten Kliniken verfügbar. Es wurde zudem nicht erfasst, inwieweit Ängste vor Nutzung und Beübung der Extremität vorlagen. Solche könnten die Funktion nach 1 Jahr negativ beeinflusst haben. Auch erfolgte keine Erfassung etwaiger psychologischer Interventionen, die entsprechend eine Bewegungsangst positiv beeinflusst haben könnten [6].

Die Anwendung invasiver Techniken bei 13 von 40 Fällen ist als hoch einzustufen. Dies könnte durch einen Selektions-Bias in Fällen mit schwerer Symptomausprägung erklärt werden. Eine objektivere Einstufung des Schweregrads mit validiertem Untersuchungstool wäre zur besseren Vergleichbarkeit hilfreich gewesen. Der inzwischen validierte CRPS Severity Score (CSS; [13]) lag zu Beginn der Studie noch nicht vor. Die Daten konnten nicht nachträglich auf den CSS übertragen werden, da nicht explizit zwischen berichteten und mittels Untersuchung erhobenen Kriterien unterschieden wurde. In einer kleineren Studie mit 33 Patient*innen war die kontinuierliche Stellatum- bzw. infraklavikuläre Plexusblockade mittels Katheter vergleichbar effektiv [27]. Da keine Kontrollgruppe ohne invasive Techniken untersucht wurde, war ein Vergleich zur konservativen Therapie nicht möglich. Anhand der in unserer Studie erhobenen Daten lassen sich aufgrund der geringen Fallzahl und zudem unterschiedlicher angewandter Techniken keine validen Aussagen zu invasiven Verfahren bezogen auf eine mögliche Verbesserung des Outcomes machen, insbesondere auch nicht hinsichtlich Vorteilen bestimmter Prozeduren. Betrachtet man die invasiven Verfahren als Gesamtheit erwies sich die Effektivität der invasiven Maßnahmen bzgl. der Outcomeparameter dieser Studie als gering. Auffällig ist jedoch, dass die Patient*innen, die invasiv behandelt wurden, bezüglich ihrer Funktionalität von ärztlicher Seite zu Behandlungsbeginn signifikant schlechter eingeschätzt wurden als die Patient*innen, die in der Folge rein konservativ behandelt wurden. Zudem gaben diese eine leicht, jedoch nicht signifikant höhere Schmerzstärke an. Beides mag die Indikationsstellung für invasive Maßnahmen beeinflusst haben. Nach einem Jahr war das Outcome beider Gruppen aber nicht unterschiedlich, d. h. die invasiv behandelten Patienten machten aus Sicht der Ärzte einen relativ größeren Behandlungsfortschritt. Interpretiert man diesen Befund, so könnte einerseits ein Bias bei den behandelnden Ärzt*innen bestanden haben, da die schmerzbezogenen Disability-Punkte nach von Korff zu Beginn nicht unterschiedlich waren, sondern eben nur die ärztlich bewertete, generellere Funktionsbeeinträchtigung. Andererseits könnte aber auch tatsächlich ein therapeutischer Effekt der invasiven Verfahren vorliegen. Während die Leitlinienempfehlungen von 2008 [3] im Einzelfall bei gravierender Schmerzsymptomatik invasive Techniken inklusive Regionalanästhesieverfahren als Option nannten, sind die aktuellen Leitlinien von 2018 [5] diesbezüglich restriktiver, wenngleich Serien an Sympathikusblockaden in spezialisierten Zentren als Option genannt werden. Mittels ultraschallgestützter Ganglion-stellatum-Blockaden bei CRPS konnte auch in einer neueren Studie eine signifikante Schmerzverbesserung gezeigt werden, wobei allerdings die Funktionalität nicht untersucht wurde [1]. Die Autoren der 2022 publizierten 5. Auflage der Leitlinien der Reflex Sympathetic Dystrophy Syndrome Association (RSDSA; [14]) empfehlen dagegen weiterhin v. a. bei Scheitern von Vortherapien und ausgeprägten Schmerzen invasive Techniken als Option. Zur Klärung eines positiven Einflusses invasiver Verfahren (insbesondere auch von Katheterverfahren) auf das CRPS-Outcome sind weitere Untersuchungen notwendig.

Generell sind Vergleiche zu anderen Outcomestudien bei CRPS schwierig. Grieve et al. [9] haben 2016 analysiert, welche Messgrößen für das Outcome bei Studien zu CRPS erhoben wurden. Sie identifizierten 68 verschiedene Kriterien von der visuellen Analogskala bis hin zu differenzierten Fragebögen, wobei nur fünf Tools speziell für CRPS validiert waren [7, 20–23]. Vier weitere Tools waren speziell adaptiert an CRPS [8, 18, 19, 28]. Die Vergleichbarkeit von Studien ist z. B. durch unterschiedliche Nachbeobachtungzeiträume erschwert. Ein Beobachtungsintervall von mindestens einem Jahr ist aus unserer Sicht zur Beurteilung notwendig, um aufgrund der oftmals nur langsamen Fortschritte eine Aussage hinsichtlich des langfristigen Outcomes treffen zu können. Ein neueres Review, das 22 Studien zum Outcome erfasste, ergab, dass 51–89 % der Patient*innen nach ≥ 1 Jahr noch Schmerzen und/oder Bewegungsstörungen hatten [15]. In unserer Untersuchung fanden sich ähnliche Ergebnisse. Nach einem Jahr beklagten 60 % Schmerzen, jedoch gaben 38 % die maximale Schmerzintensität weiterhin mit mittlerer Stärke oder darüber an (VAS_max_ ≥ 4). Aus ärztlicher Sicht fand sich nach 1 Jahr eine Funktionseinschränkung bei 52,5 % der Fälle, bei 57 % von diesen allerdings nur in leichtem Ausmaß. Dagegen ergab sich nach von Korff keine bzw. eine minimale Beeinträchtigung bei 70 % der Betroffenen. Zumindest teilweise könnten die Unterschiede durch die allgemeiner gefasste ärztliche Einschätzung der Funktionsbeeinträchtigung gegenüber der schmerzbezogenen Einschränkung nach von Korff erklärt werden. Der Fokus auf die Funktionsbeeinträchtigung und weniger auf den Schmerz könnte auch erklären, dass bei Fällen invasiver Therapien die Behandler die Funktion deutlich schlechter einschätzten als anhand der Selbstangaben nach von Korff ermittelt. Um Outcomestudien zu CRPS vergleichbarer zu machen, wurde in einem mehrstufigen Prozess der Outcomedatensatz COMPACT (Core Outcome Measurement set for complex regional PAin syndrome Clinical sTudies) entwickelt, der bei zukünftigen Studien zum Einsatz kommen sollte [10]. Neben der klinischen Erhebung der Erkrankungsschwere mittels CSS enthält dieser Datensatz Fragebögen zu Schmerz, zu Möglichkeiten zur Teilhabe sowie zu Aspekten in Bezug auf physische, emotionale und psychische Funktion bzw. Beeinträchtigung.

In Fällen mit sehr langer Krankheitsdauer bis zur Diagnosestellung muss eine bereits eingetretene Schmerzchronifizierung unterstellt werden. Ein später Behandlungsbeginn korrelierte in dieser Untersuchung auch mit einem schlechteren Outcome bzgl. Schmerzen und der Von-Korff-Disability-Punkte, nicht jedoch nach ärztlicher Einschätzung. Die Leitlinien empfehlen entsprechend eine frühzeitige Diagnosestellung und Therapie in einem interdisziplinären Setting, da das Outcome wesentlich davon abhängt [5].

Die Arbeitsunfähigkeit nach 1 Jahr war mit über 46 % der zuvor Berufstätigen in unserer Studie höher als im Review von Johnson et al. [15], die Werte zwischen 30 und 40 % ermitteln konnten. Neben einem möglichen Selektions-Bias hinsichtlich schwerer Fälle dürfte das höhere Alter unserer Stichprobe eine Rolle gespielt haben, was eine Reintegration ins Arbeitsleben auch bei geringerer Beeinträchtigung erschwert haben könnte. Generell spricht aber die hohe Anzahl langfristig beeinträchtigter CRPS-Patient*innen für die Anbindung an Zentren mit entsprechender Expertise in der Behandlung dieses komplexen Krankheitsbilds.

## Limitationen

Ein Einflussfaktor auf die Ergebnisse der vorliegenden Untersuchung könnte die Patientenselektion sein, da aufgrund der beschriebenen Rahmenbedingungen nicht gewährleistet war, dass sämtliche konsekutiven Patient*innen mit neu gestellter CRPS-Diagnose eingeschlossen wurden. Dies könnte zu einem bevorzugten Einschluss besonders schwerer Fälle geführt haben, was wiederum die Therapieauswahl und das Outcome beeinflusst haben könnte.

Grundlage der Therapien in dieser Studie waren die Leitlinien der DGN von 2008 [3]. Seitdem sind diese zweimal überarbeitet worden. Somit sind diese Ergebnisse nur eingeschränkt auf die aktuellen Leitlinien [5] übertragbar. Jedoch sind die Unterschiede überschaubar: Vor allem werden Bisphosphonate inzwischen nicht nur nach Frakturen alternativ zu Glukokortikoiden empfohlen. In dieser Studie kamen in 11 Fällen keine der Substanzen zum Einsatz, wobei das Outcome dieser Subgruppe tendenziell sogar besser war. Davon waren in 8 Fällen Frakturen Auslöser des CRPS und bereits in der alten Leitlinie hätten Bisphosphonate bei entsprechend entzündlicher Komponente zum Einsatz kommen können. Während somit auch nach den neuen Leitlinien kein wesentlich anderes Therapieverhalten zu erwarten gewesen wäre, ist ein behandlerspezifischer Einfluss eher anzunehmen. So betrug die Anwendungsrate bei Glukokortikoiden und/oder Bisphosphonaten in den vier Zentren zwischen 20 und 100 %, was vermutlich nur zum Teil durch patientenspezifische Gründe erklärt werden kann. In dieser Studie wurde zwar eine leitliniengerechte Therapie als Basis vorgegeben, jedoch sollte die Einschätzung aus Behandlersicht für die Therapieentscheidungen entscheidend sein. Während dies zwar möglicherweise Einfluss auf das Ergebnis hatte, spiegelt es aber natürlich die Situation unter den Real-life-Bedingungen des klinischen Alltags wider.

## Fazit für die Praxis

- Im klinischen Alltag ist mit einer leitliniengerechten Therapie eine effektive Behandlung eines CRPS erreichbar.
- Der Einfluss invasiver Therapien auf das langfristige Outcome bei CRPS ist ungeklärt. Weitere Studien sollten sich dabei insbesondere auch auf die Funktionalität fokussieren.
- COMPACT (Core Outcome Measurement set for complex regional PAin syndrome Clinical sTudies) soll das Outcome bei Studien vergleichbarer machen und sollte in zukünftigen Studien zum Einsatz kommen.
- Eine frühzeitige Diagnosestellung und somit ein frühzeitiger Therapiebeginn sind als essenziell für das Outcome anzusehen, sodass Verdachtsfälle auf ein CRPS zügig Einrichtungen mit entsprechender Expertise zugeführt werden sollten.

## Supplementary Information

CRPS-Erfassungs- und -Behandlungsbogen

## Data Availability

Die Daten können auf Anfrage beim korrespondierenden Autor angefordert werden.
