# Supplementary material for: Prospective, multicenter study of the outcome of complex regional pain syndrome after 12 months
Source: Schmerz. 2024 Sep 28;40(3):212–9. [Article in German] doi: 10.1007/s00482-024-00837-7 (PMC13272273; doi:10.1007/s00482-024-00837-7)
Supplement: Supplementary file 1 — CRPS-Erfassungs- und -Behandlungsbogen [file 482_2024_837_MOESM1_ESM.pdf]

Patientenetikett

CRPS Erfassungs- und Behandlungsbogen

CRPS I oder CRPS II

Anamnese:

- Spontan aufgetreten, wann:__________________
- Durch folgende Verletzung, wann:

  ____
- Nach folgender OP,

  wann:____
- Ort:____

Erstuntersuchung: Datum:_______________

- Rötung, livide Verfärbung: schwach, mittel, stark
- Schwellung: schwach, mittel, stark. Haut glänzend ja-nein
- Temperaturdifferenz ja/ nein, betroffene Extremität kälter/wärmer
- Schwitzen symmetrisch/unilateral, betroffene Extremität mehr, weniger
- Haut, Haarwachstum, Nägel atrophisch leicht, mittel, stark, Muskelatrophie leicht, mittel, stark
- Schmerz:
  - VAS mean in 24h (0-10) _______VAS max (0-10)___________
  - Einschießend oder elektrisierend ja/nein
  - Brennend ja/nein
  - attackenförmig ja/ nein
  - Allodynie ja/ nein
- Hyp- Dysästhesie ja/nein
- Neglect leicht, mittel, schwer ausgeprägt
- Tremor leicht, mittel, schwer ausgeprägt
- Andere neurologische Symptomatik ja/nein, welche_________________________________
- Funktionseinschränkung:
  - Keine
  - Leichte Einschränkung, voll berufs- alltagstauglich
  - Deutliche Einschränkung, deutliche Einschränkung in Beruf-Alltag
  - Schwere Einschränkung, auf Hilfe im Alltag angewiesen.

Aparative Untersuchungen:

- Röntgen, wann, Befund

- 3 Phasen Skelettszinthi, wann, Befund

Therapie: NSAR

Datum von-bis					
Medikament					
Stärke					

WHO II oder III

Datum von-bis					
Medikament					
Stärke					

Koanalgetika

Datum von-bis					
Medikament					
Stärke					
Datum von-bis					
Medikament					
Stärke					

Corticoide

Datum von-bis					
Medikament					
Stärke					

Bisphosphonate

Datum von-bis					
Medikament					
Stärke					

Lymphdrainage von-bis:______________________________

Passive KG von-bis:______________________________

Aktive KG von-bis:______________________________

Ergotherapie von-bis:______________________________

Spiegeltherapie von-bis:______________________________

Sympathikusblockade:

Datum					
Art					
Datum					
Art					
Datum					
Art					

Untersuchung nach 1 Monat: Datum:_______________

- Rötung, livide Verfärbung: schwach, mittel, stark
- Schwellung: schwach, mittel, stark. Haut glänzend ja-nein
- Temperaturdifferenz ja/ nein, betroffene Extremität kälter/wärmer
- Schwitzen symmetrisch/unilateral, betroffene Extremität mehr, weniger
- Haut, Haarwachstum, Nägel atrophisch leicht, mittel, stark, Muskelatrophie leicht, mittel, stark
- Schmerz:
    - VAS mean in 24h (0-10) _______VAS max (0-10)___________
    - Einschießend oder elektrisierend ja/nein
    - Brennend ja/nein
    - attackenförmig ja/ nein
    - Allodynie ja/ nein
- Hyp- Dysästhesie ja/nein
- Neglect leicht, mittel, schwer ausgeprägt
- Tremor leicht, mittel, schwer ausgeprägt
- Andere neurologische Symptomatik ja/nein, welche_________________________________
- Funktionseinschränkung:
    - Keine
    - Leichte Einschränkung, voll berufs- alltagstauglich
    - Deutliche Einschränkung, deutliche Einschränkung in Beruf-Alltag
    - Schwere Einschränkung, auf Hilfe im Alltag angewiesen.

Untersuchung nach 2 Monaten: Datum:_______________

- Rötung, livide Verfärbung: schwach, mittel, stark
- Schwellung: schwach, mittel, stark. Haut glänzend ja-nein
- Temperaturdifferenz ja/ nein, betroffene Extremität kälter/wärmer
- Schwitzen symmetrisch/unilateral, betroffene Extremität mehr, weniger
- Haut, Haarwachstum, Nägel atrophisch leicht, mittel, stark, Muskelatrophie leicht, mittel, stark
- Schmerz:
    - VAS mean in 24h (0-10) _______VAS max (0-10)___________
    - Einschießend oder elektrisierend ja/nein
    - Brennend ja/nein
    - attackenförmig ja/ nein
    - Allodynie ja/ nein
- Hyp- Dysästhesie ja/nein
- Neglect leicht, mittel, schwer ausgeprägt
- Tremor leicht, mittel, schwer ausgeprägt
- Andere neurologische Symptomatik ja/nein, welche_________________________________
- Funktionseinschränkung:
    - Keine
    - Leichte Einschränkung, voll berufs- alltagstauglich
    - Deutliche Einschränkung, deutliche Einschränkung in Beruf-Alltag
    - Schwere Einschränkung, auf Hilfe im Alltag angewiesen.

Untersuchung nach 1 Jahr: Datum:_______________

- Rötung, livide Verfärbung: schwach, mittel, stark
- Schwellung: schwach, mittel, stark. Haut glänzend ja-nein
- Temperaturdifferenz ja/ nein, betroffene Extremität kälter/wärmer
- Schwitzen symmetrisch/unilateral, betroffene Extremität mehr, weniger
- Haut, Haarwachstum, Nägel atrophisch leicht, mittel, stark, Muskelatrophie leicht, mittel, stark
- Schmerz:
    - VAS mean in 24h (0-10) _______VAS max (0-10)___________
    - Einschießend oder elektrisierend ja/nein
    - Brennend ja/nein
    - attackenförmig ja/ nein
    - Allodynie ja/ nein
- Hyp- Dysästhesie ja/nein
- Neglect leicht, mittel, schwer ausgeprägt
- Tremor leicht, mittel, schwer ausgeprägt
- Andere neurologische Symptomatik ja/nein, welche_________________________________
- Funktionseinschränkung:
    - Keine
    - Leichte Einschränkung, voll berufs- alltagstauglich
    - Deutliche Einschränkung, deutliche Einschränkung in Beruf-Alltag
    - Schwere Einschränkung, auf Hilfe im Alltag angewiesen.
